# Supplementary material for: MetaRibo-Seq measures translation in microbiomes
Source: Nat Commun. 2020 Jun 29;11:3268. doi: 10.1038/s41467-020-17081-z (PMC7324362; doi:10.1038/s41467-020-17081-z)
Supplement: Supplementary file 10 — Supplementary Data 7 [file 41467_2020_17081_MOESM10_ESM.zip › File2/Confidence_High_Taxonomy/133022_out.krona.html]

Javascript must be enabled to view this page.

members
magnitude
magnitudeUnassigned
count
unassigned
taxon
rank

133022\_out

34

superkingdom
2
34

34
phylum
976

200643
class
34

order
171549
34

34
171552
family

34
genus
838

species
2292365
1

SRS077641\_contig\_number\_2752

30

SRS012916\_contig\_number\_contig-100\_22909.22909SRS012969\_contig\_number\_contig-100\_1945.225164SRS012989\_contig\_number\_contig-100\_45.45.45SRS015794\_contig\_number\_7865SRS018888\_contig\_number\_contig-100\_1228.46468SRS019808\_contig\_number\_20354SRS023526\_contig\_number\_38829SRS043841\_contig\_number\_contig-100\_892.76923SRS044535\_contig\_number\_9593SRS045713\_contig\_number\_21384SRS045713\_contig\_number\_contig-100\_3944.127174SRS047433\_contig\_number\_contig-100\_889.116661SRS049959\_contig\_number\_27655SRS049959\_contig\_number\_52692SRS050752\_contig\_number\_11879SRS053398\_contig\_number\_contig-100\_1595.160136SRS064757\_contig\_number\_20053SRS074586\_contig\_number\_1243SRS077849\_contig\_number\_contig-100\_1023.181614SRS103987\_contig\_number\_28210SRS104036\_contig\_number\_6493SRS1041137\_contig\_number\_6948SRS104327\_contig\_number\_contig-100\_1059.43592SRS104912\_contig\_number\_20264SRS143181\_contig\_number\_2382SRS144506\_contig\_number\_contig-100\_5404.286690SRS144603\_contig\_number\_contig-100\_1887.100736SRS147614\_contig\_number\_1634SRS148253\_contig\_number\_4774SRS150029\_contig\_number\_19955
species
165179


SRS012849\_contig\_number\_26186SRS043667\_contig\_number\_6406SRS144537\_contig\_number\_6276
3
1947118
species
